# Alterations of choroidal circulation and choroidal thickness before and after chemoradiotherapy in a case of metastatic choroidal tumor

**DOI:** 10.1186/s12886-023-03026-9

**Published:** 2023-06-13

**Authors:** Mizuho Mitamura, Satoru Kase, Kiriko Hirooka, Susumu Ishida

**Affiliations:** Department of Ophthalmology, Faculty of Medicine and Graduate School of Medicine, Hokkaido University, N-15, W-7, Kita-Ku, Sapporo, 060-8638 Japan

**Keywords:** Metastatic choroidal tumor, Laser speckle flowgraphy, Optical coherence tomography, Central choroidal thickness

## Abstract

**Background:**

Metastatic choroidal tumors are hematogenous intraocular metastases of malignant tumors in systemic organs; however, the details of choroidal circulation and morphological changes in the choroid are unknown. The aim of this study is to present a case of metastatic choroidal tumor and examine laser speckle flowgraphy (LSFG)-based choroidal circulation and central choroidal thickness (CCT) before and after chemoradiotherapy.

**Case presentation:**

A 66-year-old woman with a medical history of breast cancer 16 years ago was referred to our department struggling with blurred vision in her right eye. At the time of initial examination, her best-corrected visual acuity (BCVA) was 0.4 oculus dexter (OD) and 0.9 oculus sinister. Fundus revealed a yellowish-white choroidal elevated lesion measuring 8 papillary diameters with serous retinal detachment (SRD) in the posterior pole. Fluorescein angiography showed diffuse hyperfluorescence and fluorescent leakage due to SRD, and indocyanine green angiography demonstrated no abnormalities in the macula but hypofluorescence in the center of the tumor. Based on these clinical findings, she was diagnosed with metastatic choroidal tumor. After chemoradiotherapy, the metastatic choroidal tumor became scarred, and SRD disappeared. The rate of changes in macular blood flows assessed by mean blur rate on LSFG and CCT of her right eye were 33.8 and 32.8% decrease at 5 months after the initial visit, respectively. BCVA was 0.5 OD 27 months after the initial examination.

**Conclusion:**

Chemoradiotherapy resulted in regression of the metastatic choroidal tumor and disappearance of SRD, with a decrease in central choroidal blood flow and CCT. The choroidal blood flow on LSFG could reflect an increased oxygen demand by cancer cells invading the choroid and substantial blood supply.

## Background

Metastatic uveal tumors are hematogenous intraocular metastases of malignant tumors in systemic organs, often emerging in the choroid [1]. In the entire group of 420 patients, the most common primary site of the uveal metastasis was breast (47%), followed by the lung (21%), gastrointestinal tract (4%), kidney (2%), skin (2%), prostate (2%), and other cancers (4%) [1]. Choroidal metastases are generally characterized by yellow subretinal masses (94%) with subretinal fluid (73%) [1]. Optical coherence tomography (OCT) is likely to depict the irregularity of the anterior surface of the tumor [2], with thickening of the retinal pigment epithelium along with overlying the subretinal fluid [3]. Fluorescein angiography (FA) typically shows a hypofluorescent pattern in the early phase and slower hyperfluorescence than most choroidal melanomas in the late phase [4]. Indocyanine green angiography (ICGA) usually appears hypofluorescent at all phases [5]. These multimodal imaging studies suggest that choroidal circulation may be compromised in metastatic choroidal tumors due to subretinal fluids, choroidal invasion and embolization of tumor cells.

Laser speckle flowography (LSFG) is a blood flow imaging system that uses laser scattering to visualize fundus circulation in two dimensions, which enables to noninvasively investigate intraocular circulation in various patients. We have analyzed retino-choroidal circulation of intraocular tumor(-like) lesions such as optic disc melanocytoma [6], choroidal macrovessel [7], sclerochoroidal calcification [8], juxtapapillary retinal capillary hemangioblastoma [9], choroidal lymphoma [10], leukemic retinopathy [11, 12], and radiation retinopathy for choroidal melanoma [13]. However, the details of choroidal circulation and morphological changes in metastatic choroidal tumor before and after treatment are unknown.

We herein present a case of metastatic choroidal tumor treated with chemoradiotherapy and examine choroidal circulatory and central choroidal thickness (CCT) changes using LSFG and swept source (SS)-OCT, respectively.

## Case presentation

**Fig. 1:**
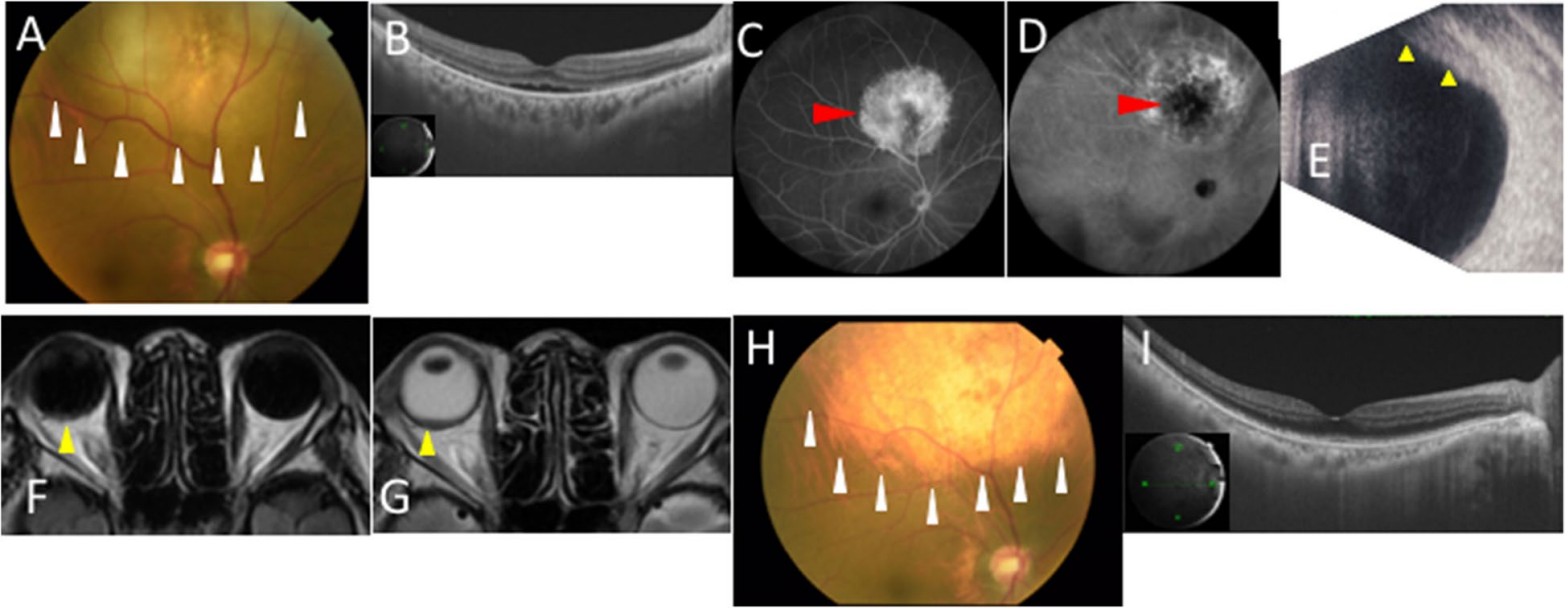
Initial findings on color fundus photography (CFP), swept-source optical coherence tomography (SS-OCT), fluorescence angiography (FA), indocyanine green angiography (ICGA), B-mode echography, and orbital magnetic resonance imaging (MRI) in the present case with metastatic choroidal tumor. **A** CFP showed a yellowish-white choroidal elevated lesion of 8 papillary diameters in the posterior pole of the fundus oculus dexter (OD) (white arrowheads). **B** SS-OCT at horizontal scans through fovea showed serous retinal detachment (SRD) from the macula to the choroidal mass area OD. **C** FA showed the hyperfluorescent area in the late phase OD (red arrowhead). **D** ICGA showed that the center of the tumor was hypofluorescent (red arrowhead), while the periphery of the tumor was hyperfluorescent at the late phase OD. **E** B-mode echography revealed a flat elevated lesion OD (yellow arrowheads). **F** T1-weighted MRI image showed an iso-signal flat elevated lesion within the right eye (yellow arrowhead). **G** T2-weighted MRI image showed a low-signal flat elevated lesion within the right eye (yellow arrowhead). **H** CFP showed resolution of elevated lesions and brownish retina OD 27 months after the initial visit (white arrowheads). **I** SS-OCT at horizontal scans through fovea revealed disappearance of SRD OD 27 months after the initial visit

A 66-year-old woman complained of blurred vision in her right eye and was referred to our hospital because of a choroidal elevated lesion at the right superior fundus. She had a history of left mastectomy due to left breast cancer 16 years ago. There were no special notes on family history. At the time of initial examination, her best-corrected visual acuity (BCVA) was 0.4 oculus dexter (OD) and 0.9 oculus sinister (OS) with mild myopia and astigmatism, and her intraocular pressure was normal oculi uterque (OU). Slit-lamp microscopy did not detect any findings OU. Color fundus photography (CFP) showed a yellowish-white choroidal elevated lesion measuring 8 papillary diameters in the posterior pole of the fundus (Fig. 1A). SS-OCT showed serous retinal detachment (SRD) localized from the macula to the choroidal elevation (Fig. 1B). Moreover, SS-OCT depicted the irregularity of the anterior surface of the tumor with high reflective material deposits beneath the retina. FA showed diffuse hyperfluorescence in the tumor area in the early phase, and the enhanced hyperfluorescent area and fluorescence leakage due to SRD in the late phase (Fig. 1C). ICGA demonstrated no obvious fluorescence blockage or abnormal choroidal vascularity but hypofluorescence in the center of the tumor in the early phase. The center of the tumor remained hypofluorescent, while the periphery of the tumor was hyperfluorescent in the late phase (Fig. 1D). B-mode echography revealed a flat elevated lesion (Fig. 1E). The tumor showed iso-intensity and low intensity on T1-weighted and T2-weighted images on orbital magnetic resonance imaging, respectively (Fig. 1F and G).

Based on these ophthalmologic findings and a medical history of breast cancer, she was diagnosed with metastatic choroidal tumor. The systemic examination revealed multiple organ metastases including the bone, lung, liver, and mediastinal lymph node. Biopsy was conducted from a mediastinal lymph node, the histopathology of which was consistent with a pathological diagnosis of adenocarcinoma as a metastasis of breast cancer. Immunohistochemically, estrogen receptor was positive, progesterone receptor was marginally positive, and human epidermal growth factor receptor type 2 was positive in tumor cells. One month after the initial visit, radiation therapy (30 Gy/10 fraction) was performed for the right eye. Two months after the initial visit, combined chemotherapy (veltuzumab + trastuzumab + docetaxel) was started. Five months after the initial visit, her BCVA was 0.9, metastatic choroidal tumor became scarred, and SRD disappeared on OCT. Twenty-seven months after the initial visit, her BCVA was 0.5, and CFP showed resolution of elevated lesions and scar formation in the retina where the tumor had existed OD (Fig. 1H). SS-OCT showed disappearance of SRD at the macula and around the superior arcade vessels (Fig. 1I). The patients did not show any findings including non-perfusion areas, retinal hemorrhage, or soft exudates, suggestive of radiation retinopathy during the course of the study. The institutional review board of Hokkaido University waived ethical assessment of this clinical study because of a single case report with a non-invasive study. This study adhered to the tenets of Declaration of Helsinki.

**Fig. 2:**
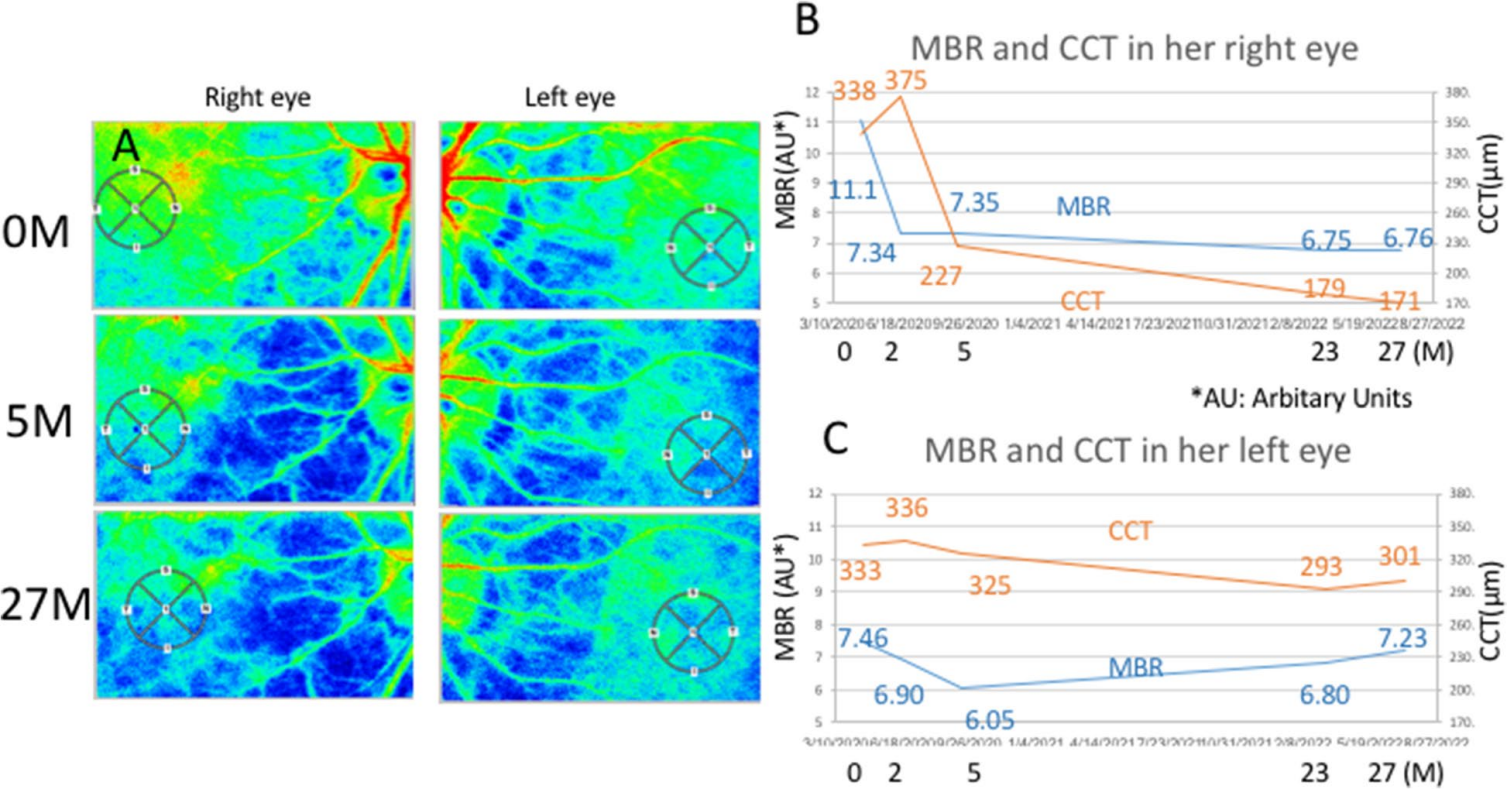
Laser speckle flowgraphy (LSFG) images, and mean blur rate (MBR) values and the central choroidal thickness (CCT) in a case of metastatic choroidal tumor. **A** LSFG images at initial visit, 5 months, and 27 months after the initial visit. A circle of 750 μm in diameter at the fovea was defined as the region of interest on LSFG. Compared to the initial examination, both eyes had relatively cold-color signal at 5 and 27 months. **B** MBR decreased after initiation of chemoradiotherapy and remained almost unchanged after remission OD. CCT increased temporarily after the start of chemotherapy but decreased after remission OD. **C** MBR gradually decreased from the time of initial diagnosis after the start of chemotherapy, and later increased in her left eye. CCT did not change at all during the course of the study

This study further analyzed choroidal circulatory and the CCT changes. First, the changes in choroidal blood flows before and after chemoradiotherapy were evaluated using LSFG. Relative values of the blood flow were determined as mean blur rate (MBR) based on quantitative measurements of blood flow velocity by LSFG software (LSFG-NAVI^R^, version 3.1.39.2, Softcare Ltd., Fukuoka, Japan) according to previous reports [12]. Before the examination, the pupils of both eyes were completely mydriatic with 0.4% tropicamide (Santen Pharmaceutical Co., Ltd., Osaka) to obtain a mydriatic state in which the pupils of both eyes were completely devoid of light reflex. The macula in the MBR image was marked manually by one experienced operator, and the vessels were segmented using threshold values automatically defined by the system software (LSFG Analyzer, version 3.0.47.0). A circle of 750 μm diameter in the macula OU was defined as the region of interest on LSFG (Fig. 2A: small circles). Four to five consecutive measurements were taken for each circle, and the mean values were calculated and used for analysis. Ocular perfusion pressure (OPP) was calculated using the patient’s blood pressure and intraocular pressure in accordance with previous reports [12].

The MBR values OD were shown in Fig. 2B as follows: 11.1, 7.34, 7.35, 6.75, and 6.76 arbitrary units (AU) at the initial visit, 2, 5, 23, and 27 months after the initial visit, respectively. The MBR of the right eye decreased immediately after the start of treatment and remained unchanged thereafter. The rate of change assessed by MBR was 33.8% decrease OD 5 months after the initial visit (Fig. 2B). The MBR values OS were shown in Fig. 2C as follows: 7.46, 6.90, 6.05, 6.80, and 7.23 AU at the initial visit, 2, 5, 23, and 27 months after the initial visit, respectively. The rate of change assessed by MBR was temporarily 18.9% decrease OS 5 months after the treatments (Fig. 2C). OPP was 66.6, 66.1, 66.3, 65.4, and 59.2 mmHg OD and 65.6, 61.1, 61.3, 64.4, and 57.2 mmHg OS at the initial visit, 2, 5, 23, and 27 months after the initial visit, respectively, proving no significant changes in both eyes.

Next, the CCT was measured manually from the lower edge of the retinal pigment epithelium layer to the scleral border by one experienced examiner using SS-OCT (DRI OCT Triton; Topcon Inc., Tokyo, Japan). The CCT OD were 338, 375, 227, 179, and 171 µm at the initial visit, 2, 5, 23, and 27 months after the initial visit, respectively (Fig. 2B). The CCT of the right eye decreased 5 months after the initial visit and kept thinning thereafter. The CCT OS were 333, 336, 325, 293, and 301 µm at the initial visit, 2, 5, 23, and 27 months after the initial visit, respectively (Fig. 2C).

## Discussion and conclusion

In this case, the MBR was high before treatment indicating choroidal hyperperfusion in the central choroid, where SRD was present with increased CCT. Comparing the rate of change assessed by MBR between the right and left eyes, 5 months after the first visit when chemoradiotherapy was completed, the rate of decrease was 33.8% OD and 18.9% OS. In the right eye with metastatic choroidal tumor, it was suggested that choroidal blood flow may have increased before treatment. On the other hand, the MBR was low after treatment and the CCT decreased when SRD disappeared, suggesting hypoperfusion and choroidal thinning, both of which are associated with regression of the metastatic choroidal tumor.

There are at least two possible mechanisms for LSFG-based central choroidal hyperperfusion and increased CCT in SRD associated with metastatic choroidal tumor. First of all, the route of choroidal metastasis from breast cancer is hematogenous [14]; however, whether alterations of MBR and CCT are originated from tumor cell activity or SRD are unknown. According to previous reports, the changing rates of the average MBR and CCT significantly increased and decreased, respectively, with resolution of SRD in response to systemic corticosteroids in Vogt-Koyanagi-Harada disease [15]. Moreover, the retinal blood flow on the optic nerve head of the average MBR increased after vitrectomy in eyes with a rhegmatogenous retinal detachment [16]. On the other hand, in the present study, the MBR decreased at the timing of the disappearance of SRD after chemoradiotherapy for metastatic choroidal tumor. If the current data were consistent with the previous reports, resolution of SRD would theoretically increase MBR and decrease CCT, but on the contrary, the MBR decreased in this case. These results suggest that hyperperfusion in the central choroid could reflect an increased oxygen demand by cancer cells invading the choroid rather than an effect on SRD. As a result, the MBR might have decreased due to the reduced oxygen demand caused by the decrease in the number of cancer cells after remission following multidisciplinary treatment. Taken together, monitoring MBR trends may be useful in evaluating treatment response in metastatic choroidal tumors.

The other possibility of the hyperperfusion is associated with the choroidal vasculature in the metastatic tumors. Uveal metastases occur more frequently in the choroid than in the iris, possibly due to a difference in substantial blood supply via the posterior ciliary arteries [1]. Therefore, increased MBR observed in this case may have correlated with the blood supply in choroidal metastases. Previous reports indicated that hematogenous dissemination of metastases from distant organs was usually assumed to result in a high-flow choroidal vasculature with metastatic disease [4]. The choroid, as the metastatic site, provides a vascular corridor for tumor embolization sequestration and a receptive environment for growth [4]. In this case, ICGA showed that the center of the tumor was hypofluorescent, while the periphery of the tumor was relatively hyperfluorescent in the late phase, which may indicate tumor embolization in the central part of the lesion. Moreover, MBR on LSFG showed a high value before treatment in the right eye, indicating increased choroidal blood flow with tumor invasion. Evaluation of MBR in the central choroid “before choroidal metastasis” in breast cancer patients would clarify the pathophysiological importance of hyperperfusion in the central choroid in the future.

Her left eye with free of tumor cells also showed no abnormalities of the fundus during the follow-up periods, but marginal decrease in MBR between 2 and 5 months after the initial visit. These results suggest potential impacts on the chemotherapy drugs involving MBR in both eyes. However, there are no reports regarding the effect of the chemotherapy drugs administered to our patient on choroidal thickness or choroidal circulation. In other words, although chemotherapy may potentially affect the choroidal circulation, the decrease in the MBR and CCT of the right eye could be more likely to result from the choroidal tumor regression in response to treatment than subclinical changes by the chemotherapy.

There are limitations in this study. First, this is a single case study, and further studies are needed to determine the trend of changes in MBR and CCT values in metastatic choroidal tumors. Second, this case showed SRD in the macula before treatment, which may affect acquisition of the MBR value. LSFG utilizes a wavelength of 830 nm, similar to ICGA, and can adequately evaluate blood flow mainly in the deep choroid [17]. In a report of LSFG in acute central serous chorioretinopathy (CSC), both area with and without SRD in the macula showed similar changes at the time of CSC regression [18]. Therefore, we consider the effect of SRD on MBR measurements to be minimal in this study, but further validation is needed. Forth, in this case, the tumor was relatively too peripheral in the fundus to obtain reliable LSFG images and MBR values at the tumor site due to limitation of fixation. Finally, the precise optic papillary blood flows were not available on LSFG in this study; therefore, further studies are needed to elucidate the potential effects of systemic chemotherapy on the optic papillary blood flows.

In conclusion, multidisciplinary treatment resulted in regression of the metastatic choroidal tumor and disappearance of SRD, together with decreased choroidal blood flow and CCT. The choroidal blood flow in metastatic choroidal tumors could reflect an increased oxygen demand by cancer cells and substantial blood supply in the choroid.

## Data Availability

Not applicable.

## Abbreviations

CCT: Central choroidal thickness
LSFG: Laser speckle flowgraphy
BCVA: Best-corrected visual acuity
OD: Oculus dexter
OS: Oculus sinister
OU: Oculi uterque
SS-OCT: Swept-source optical coherence tomography
FA: Fluorescence angiography
ICGA: Indocyanine green angiography
MRI: Magnetic resonance imaging
SRD: Serous retinal detachment
MBR: Mean blur rate
AU: Arbitrary units
OPP: Ocular perfusion pressure
CSC: Central serous chorioretinopathy
